# Ghanaian parents' perceptions of pre and postnatal nutrient supplements and their effects

**DOI:** 10.1111/mcn.12608

**Published:** 2018-04-15

**Authors:** Katherine P. Adams, Harriet Okronipa, Seth Adu‐Afarwuah, Mary Arimond, Sika Kumordzie, Brietta M. Oaks, Maku E. Ocansey, Rebecca R. Young, Stephen A. Vosti, Kathryn G. Dewey

**Affiliations:** ^1^ Program in International and Community Nutrition, Department of Nutrition University of California, Davis Davis California USA; ^2^ Department of Nutrition University of California, Davis Davis California USA; ^3^ Department of Nutrition and Food Science University of Ghana Accra Ghana; ^4^ Intake—Center for Dietary Assessment FHI 360 Washington District of Columbia USA; ^5^ Department of Nutrition and Food Sciences University of Rhode Island Kingston Rhode Island USA; ^6^ Department of Agricultural and Resource Economics University of California, Davis Davis California USA

**Keywords:** child nutrition, Ghana, maternal nutrition, multiple micronutrients, perceptions, small‐quantity lipid‐based nutrient supplements

## Abstract

Small‐quantity lipid‐based nutrient supplements (SQ‐LNS) have been studied in efficacy and effectiveness trials, but little is known about how parents perceive the products and their effects. In a randomised trial in Ghana, efficacy of SQ‐LNS provided to women during pregnancy and the first 6 months postpartum and to their children from 6 to 18 months of age was assessed by comparison with iron‐folic acid (IFA) capsules and multiple micronutrient (MMN) capsules provided to women. In a follow‐up study conducted when the index children from the original trial were between 4 and 6 years of age, we used survey‐based methods to assess retrospective and current parental perceptions of nutrient supplements generally and of SQ‐LNS and their effects compared with perceptions IFA and MMN capsules. Most parents perceived that the assigned supplements (SQ‐LNS, IFA, or MMN) positively impacted the mother during pregnancy (approximately 89% of both mothers and fathers) and during lactation (84% of mothers and 86% of fathers). Almost all (≥90%) of mothers and fathers perceived that the assigned supplement positively impacted the index child and expected continued positive impacts on the child's health and human capital into the future. A smaller percentage of parents perceived negative impacts of the supplements (7%–17% of mothers and 4%–12% of fathers). Perceptions of positive impacts and of negative impacts did not differ by intervention group. The results suggest that similar populations would likely be receptive to programs to deliver SQ‐LNS or micronutrient capsules.

**Key messages**

- There is limited evidence on how parents perceive small‐quantity lipid‐based nutrient supplements and their effects.
- In a follow‐up study in Ghana, most parents perceived that the assigned supplements (iron‐folic acid capsules for mothers, multiple micronutrient capsules for mothers, or small‐quantity lipid‐based nutrient supplements for mothers and their infants) positively impacted both the mother and her child, whereas perceptions of negative effects were uncommon.
- Perceptions did not differ by intervention group.
- Accounting for both positive and negative perceptions of nutrient supplements in the design and management of programs to deliver them may help promote acceptability and compliance in similar populations.

## 1 INTRODUCTION

Small‐quantity lipid‐based nutrient supplements (SQ‐LNS) were designed to prevent undernutrition during the critical “first 1,000 days”, from conception to age two. In addition to a range of micronutrients, SQ‐LNS also enrich the diets of pregnant and lactating women and of young children with some key macronutrients, including essential fatty acids. Over the past decade, studies in sub‐Saharan Africa (Adu‐Afarwuah et al., 2016; Adu‐Afarwuah et al., 2015; Adu‐Afarwuah, Lartey, Zeilani, & Dewey, 2011; Ashorn, Alho, Ashorn, Cheung, Dewey, Gondwe, et al., 2015a; Ashorn, Alho, Ashorn, Cheung, Dewey, Harjunmaa, et al., 2015b; U. Ashorn et al., 2015c; Hess et al., 2015; Klevor et al., 2016; Maleta et al., 2015; Phuka et al., 2008), South Asia (Dewey et al., 2017; Mridha et al., 2016), and the Caribbean (Iannotti et al., 2014) have provided evidence on the acceptability (i.e., willingness to consume the product and/or feed it to an infant, opinions about sensory characteristics like colour, odour, and taste, etc.), efficacy, and effectiveness of SQ‐LNS (~20 g/day). However, very little is known about participant perceptions of interventions that provide SQ‐LNS and perceptions of the effects of SQ‐LNS.

Participant perceptions are an important component of the overall evidence base on SQ‐LNS and other pre and postnatal nutrient supplements. In particular, understanding how parents perceive supplements and interventions that provide them can help inform future efforts to scale‐up the delivery of SQ‐LNS or other point‐of‐use micronutrient fortified products. Also, there is evidence that parents' investments in their child's health and human capital can be influenced by an early‐life intervention that parents perceived had a positive effect on their child's health or cognitive development (Almond & Mazumder, 2013). In Tanzania, for example, children with prenatal exposure to a maternal iodine supplementation program were breastfed longer and were more likely to be vaccinated than unexposed children (Adhvaryu & Nyshadham, 2016). Knowledge about parental perceptions, then, may be important for the design and management of programs to deliver SQ‐LNS or other micronutrient supplements and for understanding the potential for supplementation to influence parents' investments in their children.

Between December 2009 and March 2014, the International Lipid‐Based Nutrient Supplement (iLiNS) Project implemented a randomised controlled trial in Ghana to test the efficacy of SQ‐LNS. The trial provided SQ‐LNS to mothers during pregnancy and the first 6 months postpartum and to their infants from 6 to 18 months of age. SQ‐LNS provided to mothers during pregnancy increased infant birth size, particularly among primiparous mothers (Adu‐Afarwuah et al., 2015), and comprehensive SQ‐LNS to mothers during pregnancy and the first 6 months postpartum and to their infants from 6 to 18 months of age increased mean attained length at 18 months (Adu‐Afarwuah et al., 2016). Child developmental outcomes at 18 months did not differ between intervention groups (Prado et al., 2016).

In this paper, using survey‐based quantitative data collected during a follow‐up study several years after the trial, we first describe mothers' and fathers' perceptions of maternal supplementation during pregnancy and lactation and perceptions of child supplementation during early childhood. This initial descriptive analysis characterizes parents' perceptions of maternal and child nutrient supplementation in a general sense rather than perceptions of specific supplements. Then, leveraging the trial's randomised design, we report on the effects of the intervention on parents' retrospective and current perceptions of maternal and child supplementation with SQ‐LNS vis‐à‐vis their perceptions of the effects of maternal supplementation with iron‐folic acid (IFA) or multiple micronutrient (MMN) capsules. Finally, we describe mothers' retrospective perceptions of their children's reactions to consuming SQ‐LNS. Because these perceptions data were collected several years after the intervention concluded, parents had time to observe how the intervention affected them and their child in both the short‐ and medium‐ terms and also to consider their perceptions of the intervention from a more detached standpoint. As such, the findings here complement assessments of perceptions performed during the intervention itself.

## 2 METHODS

### 2.1 Main trial

The main trial was conducted in several adjoining semiurban communities in the Yilo Krobo and Lower Manya Krobo districts in the Eastern Region of Ghana that were situated along a busy commercial corridor. Households in the area earned income largely via nonfarm sources, such as engagement in petty trade, the operation of shops and kiosks, and the provision of skills and services. In 2014, 17% of children under five in the Eastern Region were stunted (Ghana Statistical Service, Ghana Health Service, & ICF International, 2015).

From December 2009 through December 2011, pregnant women were recruited into the main trial on a rolling basis during routine visits to one of four main health facilities in the area. Eligible women who provided informed consent were individually randomised into one of the trial's three equally‐sized intervention groups: (a) daily IFA capsules during pregnancy, a component of the standard of antenatal care in Ghana, a low‐dose calcium placebo capsule for the first 6 months postpartum, and no infant supplementation (b) daily MMN capsules during pregnancy and the first 6 months postpartum and no infant supplementation, or (c) 20 g/day SQ‐LNS during pregnancy and the first 6 months postpartum followed by 20 g/day SQ‐LNS for the infant from 6 to 18 months of age. The IFA and MMN capsules were identical in appearance, and mothers in the IFA and MMN groups were blinded to which capsule they received. Because the micronutrients in SQ‐LNS were delivered in a food‐based paste, it was not possible to blind the lipid‐based nutrient supplements (LNS) group. The nutrient content of each of the capsules and of the SQ‐LNS product for pregnant and lactating women and the SQ‐LNS product for children are available in Table S1.

At enrolment, trained nurses told all women that because women need more vitamins and minerals when they are pregnant, they would be receiving a supplement. Nurses also gave women instructions for taking their assigned supplement and a standard minimum nutrition message reminding them to continue to consume nutritious foods whenever possible. During pregnancy, all women were visited by project staff every 2 weeks to deliver supplements and collect data on maternal morbidity and study adherence. After the birth of the infant (hereafter index child), home visits occurred weekly. When the index children were 6 months of age, all mothers were instructed on the importance of continuing to breastfeed and were provided with a minimum nutrition message reminding them to give their infants nutritious foods. The text of the instructional and minimum nutrition messages is available in Supplemental Methods 1.

The scheduled frequency of contact between study staff and the women and infants enrolled in the study as well as the content of those visits were, by design, uniform across intervention groups. However, some women received additional unscheduled visits to address difficulties with taking their assigned supplement or difficulty feeding their infants. In cases of the former, nurses worked with the women to identify the problem and suggest a solution (e.g., take the supplement at a different time of day, mix the supplement with a different food, etc.). In cases of the latter, nurses advised mothers on ways to encourage their infants to eat and reiterated the standard minimum nutrition message, noting that it can take time for infants to accept new foods.

### 2.2 Follow‐up

When the index children were between 4 and 6 years of age, we conducted a follow‐up study with the primary objective of assessing the longer term effects of SQ‐LNS on child growth and development. Several months before the start of follow‐up data collection, we attempted to reestablish contact with all households from the main trial, excluding misdiagnosed pregnancies (*n* = 5) and miscarriages (*n* = 37), to inform them about the follow‐up and to update their contact information. At the beginning of the follow‐up data collection period, which spanned January 2016 to December 2016, we visited households to provide details about the follow‐up, obtain informed consent, and collect socioeconomic data.

As a component of the follow‐up data collection activities, we used survey‐based methods to collect retrospective and current perceptions data from mothers and, separately, from fathers. Survey‐based methods were used to allow for statistical comparisons of perceptions between the intervention groups. The first set of questions asked mothers and fathers about their current perceptions of nutrient supplements in general (i.e., not supplements specific to the intervention). The next set of questions pertained specifically to the supplements received during the intervention. To ensure that parents recognized that the questions were meant to elicit both positive and negative perceptions of the assigned supplements and their effects, data collectors prefaced that set of questions with the following: “Now we would like to ask you some questions about your experiences regarding the iLiNS project. We are interested in both positive and negative experiences. Please think back to when you were a participant in this project and feel free to share with us whatever your experiences were, whether good or bad. Remember, it's ok to share both.” Then, mothers and fathers were shown a picture of SQ‐LNS and a picture of a capsule and asked to recall which supplement the mother received during the intervention. This was followed by a series of questions that assessed parents' retrospective and current perceptions of the effects of the assigned supplements (SQ‐LNS, IFA capsules, or MMN capsules) on the mother, the index child, and other household members. These questions were posed to all mothers and fathers regardless of whether or not they correctly recalled which supplement was received during the intervention. A final set of questions, posed only to mothers in the LNS group who correctly recalled that their child received SQ‐LNS during the intervention, were specific to maternal perceptions of experiences related to child consumption of SQ‐LNS.

Almost all survey questions were open ended, and data collectors were instructed to record multiple responses if a respondent provided more than one answer to a question. Two sets of questions had prespecified response options that were read out to respondents. The first was a set of questions asking mothers and fathers to project into the future and think about their expectations of the supplement's potential impact on the index child's ability to do well in school, his/her cognitive development, his/her ability to do physical work, and his/her ability to earn money. Parents were asked to choose one response among “no impact”, “positive impact”, or “negative impact”. The second was a question posed only to mothers in the LNS group asking them to recall how frequently (less than once a week, about once a week, a few times a week, or every day) their child asked for SQ‐LNS in the few weeks following the end of the intervention. All survey questions are available in Supplemental Methods 2.

The follow‐up study was approved by the ethics committees of the University of California, Davis, the Ghana Health Service, and the University of Ghana, College of Basic and Applied Sciences. The main trial was registered at clinicaltrials.gov as NCT00970866.

### 2.3 Sample size and statistical methods

The original sample size for the main trial was 1,320 women, with an equal number of women per group as described in Adu‐Afarwuah et al. (2015). At follow‐up, we attempted to include all mothers and fathers from the main trial, excluding misdiagnosed pregnancies, miscarriages, and, for the follow‐up sample of mothers, maternal deaths.

To evaluate the effect of the provision of SQ‐LNS on parental perceptions, we combined the IFA and MMN capsule groups into one group (capsules group) and compared them with the LNS group. The randomised design of the main trial ensured balance among the intervention groups. However, attrition in the follow‐up study raised the potential for nonrandom imbalance between groups at follow‐up. We evaluated the extent of this potential issue in our data in several ways. First, we used logistic regression to compare attrition between the intervention groups. We also evaluated differences in baseline characteristics between the sample that was captured at follow‐up and those who were lost to follow‐up using logistic (for dichotomous variables) and ordinary least squares (OLS) (for continuous variables) regression. Finally, we used logistic and OLS regression to evaluate balance in baseline characteristics in the follow‐up sample between the LNS group and the capsules group.

As noted, most survey questions were open ended, and data collectors were instructed to record multiple responses. We dichotomized each response, and, for perceptions analysed by group, we defined a “gatekeeper” outcome in order to limit the number of hypothesis tests (a total of 13 gatekeeper outcomes were analysed by group for mothers and for fathers). If there was a significant difference by group for the gatekeeper outcome, we proceeded to that perception's suboutcomes. For example, if the survey question was “Can you share with me any negative impact on you yourself that was due to the Nkatepa [the local name for SQ‐LNS]/capsule that you took during pregnancy?,” we created a dichotomous variable to indicate whether the respondent reported any negative impacts. Here, the gatekeeper was “The assigned supplement had a negative impact on the mother during pregnancy.” If there was a significant difference in the proportion with this perception between groups, we proceeded to evaluate whether there were group differences in specific negative impacts that were mentioned by respondents. For gatekeeper outcomes, responses of “does not know” were coded as missing.

The main analyses were conducted in Stata 14 (StataCorp) by intention‐to‐treat using logistic regression for dichotomous outcomes and ordered logit models for ordered outcomes. Hypothesis tests were two sided at the 5% level of significance. Mothers' and fathers' perceptions were analysed separately. All models were estimated twice, first adjusting for index child age at the time of the perceptions interview and then with age plus an additional set of prespecified baseline covariates. Baseline covariates were included in the fully adjusted models if they were associated with the outcome at the 10% level of significance in a bivariate analysis or if they were significantly different in the follow‐up sample compared with the lost to follow‐up sample. For the mothers' perceptions analyses, prespecified covariates were index child gender, index child birth order, maternal height, maternal age, maternal education, gender of head of household, and household electrification. Prespecified covariates for the analyses of fathers' perceptions were index child gender, index child birth order, mother's education, father's education, gender of head of household, and household electrification. For outcomes with extremely low variation in responses (i.e., ≥95% of all responses were the same), we reported percentages by group but did not analyse these outcomes for differences between groups.

Perceptions of use of supplements in general (i.e., not supplements specific to the intervention) were summarized over all mothers and over all fathers and were not analysed by intervention group. For these general perceptions and for maternal perceptions of experiences related to child consumption of SQ‐LNS (collected only from mothers who correctly recalled that their child received SQ‐LNS), responses to open‐ended questions were reported if they were mentioned by at least 5% of either mothers or fathers. Questions that were specific to child supplementation were skipped if the index child had died.

## 3 RESULTS

### 3.1 Attrition and balance

Of the 1,320 mothers who were originally randomised into the main trial, we attempted to recontact 1,273 mothers. Misdiagnosed pregnancies (*n* = 5), miscarriages (*n* = 37), and maternal deaths (n = 5) were excluded from follow‐up. We successfully recontacted and interviewed 981 mothers (rate of successful follow‐up of 77.1%). Based on the original sample of 1,320 mothers, the overall rate of attrition was 25.7%. Overall attrition was 23.9% in the LNS group and 26.6% in the capsules group and did not differ between groups (*p* = .29).

We attempted to follow up with 1,278 fathers, excluding fathers of misdiagnosed pregnancies (*n* = 5) and miscarriages (*n* = 37). Primarily because fathers were difficult to reach at home during the day, had moved out of the study area and could not be located, or had separated from the mother since the main trial, the number of fathers who were successfully interviewed (*n* = 296) was well below the number of fathers we attempted to interview. The success rate for paternal interviews, based on the originally randomised sample of 1,320, was 23.6% in the LNS group and 21.8% in the capsules group (*p* = .46). Given these low success rates, the fathers who were interviewed may represent a biased sample of all fathers and therefore all analyses of fathers' perceptions are considered exploratory.

Table 1 presents a comparison of selected baseline and index child characteristics between the maternal sample for the follow‐up and those lost to follow‐up. Compared with those lost to follow‐up, the average age of the index children at the beginning of follow‐up data collection was slightly younger among mothers in the follow‐up sample. Mothers in the follow‐up sample were also approximately a year and a half older, on average, had slightly more pregnancies prior to the index child, and were less likely to have resided in female‐headed households at baseline. The average birth weight of index children of mothers in the follow‐up sample was slightly higher than in the lost to follow‐up sample. Among mothers in the follow‐up sample, baseline and index child characteristics were balanced between the LNS and capsules groups (Table S2).

**Table 1:** Characteristics of participants in the maternal follow‐up compared with those lost to follow‐up

	Follow‐up sample		Lost to follow‐up sample		
Variable	*N*	Mean ± *SD*	*N*	Mean ± *SD*	*p* value
Index child age at start of follow‐up data collection (year)	980	4.6 ± 0.6	267	4.7 ± 0.6	.03
Index child male (%)	981	49	267	51	.52
Maternal parity at birth of index child (*n*)	981	2.3 ± 1.3	339	2.1 ± 1.2	.01
Maternal age (year)	981	27.0 ± 5.4	339	26.0 ± 5.7	.01
Maternal education (year)	978	7.4 ± 3.6	321	7.5 ± 3.9	.89
Maternal height (m)	980	1.59 ± 0.06	336	1.58 ± 0.06	.06
Head of household female (%)	976	26	319	34	.01
Household has electricity (%)	978	86	321	82	.12
Index child birth weight (g)	929	2996 ± 425	229	2926 ± 455	.03
Index child LAZ at 18 months	876	−0.82 ± 1.0	168	−0.91 ± 1.0	.31

*Note*: Values are mean ± standard deviation for continuous variables and percentage for dichotomous variables. *p* values for tests of difference in mean/percentage between follow‐up and lost to follow‐up samples from logistic (for dichotomous variables) and OLS (for continuous variables) regressions. LAZ = length‐for‐age z‐score.

### 3.2 Perceptions of use of supplements

Perceptions of the use of supplements in general (i.e., not supplements specific to the intervention) are presented for all mothers and fathers in Table 2. The most common reasons mothers gave for why women might use any supplements during pregnancy were “to get more blood”, which is how Ghanaians typically refer to the prevention of anaemia (mentioned by 57.9% of mothers), “for the growth of the baby” (53.2%), “for good energy or health” (47.1%), and “to increase maternal appetite” (23.3%). “To get more blood” was also the most common reason cited by mothers for why women might use supplements during lactation (45.4%). Mothers also mentioned that supplements might be taken during lactation to make the mother stronger (37.4%), for the baby's health (27.2%), to increase maternal appetite (24.4%), and to increase breastmilk production (16.1%). Fathers had very similar perceptions about why women might use supplements during pregnancy and lactation.

**Table 2:** Maternal and paternal perceptions of use of supplements

		Mothers		Fathers	
		*N*	Percent^1^	*N*	Percent^1^
(1) Reasons a woman might use supplements during pregnancy		980		295	
	To get more blood		57.9		50.3
	For growth of the baby		53.2		53.2
	For good health/energy		47.1		47.1
	To increase appetite		23.3		19.7
	To gain weight		4.5		6.1
(2) Reasons a woman might use supplements during lactation		978		295	
	To get more blood		45.4		53.2
	To make mother stronger		37.4		29.5
	For baby's health		27.2		31.9
	To increase appetite		24.4		17.3
	For more breastmilk		16.1		19.0
	Does not know		5.2		5.1
	No special reason		5.1		6.4
(3) Reasons a woman might not use supplements during pregnancy		979		296	
	Nausea/vomiting		24.2		19.6
	Laziness		17.0		14.2
	Does not know		16.9		12.8
	No need		16.7		19.6
	No special reason		11.0		13.5
	Too costly		6.3		8.1
	Dizziness		5.6		4.1
	Dislikes medicine/supplements		5.3		5.4
	Lack of knowledge/ignorance		1.0		6.1
(4) Reasons a woman might not use supplements during lactation		975		295	
	No need		30.3		31.5
	Does not know		25.7		22.0
	No special reason		20.3		18.6
	Too costly		9.0		8.1
	Lack of knowledge/ignorance		0.4		5.8
(5) Reasons parents might give children supplements between the ages of 6 months and 2 years		956		288	
	To increase appetite		56.4		44.8
	For good health		47.3		54.5
	To gain weight		13.7		14.6
	To be more active		11.0		6.9
	For growth		10.6		7.6
	To get more blood		9.3		6.6
	To prevent undernutrition		7.6		4.2
	Does not know		6.4		4.9
	No special reason		4.7		9.0
(6) Reasons parents might not give children supplements between the ages of 6 months and 2 years		958		288	
	No need		37.4		35.1
	No special reason		26.4		21.2
	Too costly		15.2		11.8
	Does not know		9.7		12.9
	Advice from others		2.1		7.3
(7) LNS group: Accuracy of recollection of supplement mother received during the intervention		336		104	
	Correct recollection		98.5		76.0
	Incorrect recollection		1.2		2.9
	Does not know/remember		0.3		21.2
(8) Capsules group: Accuracy of recollection of supplement mother received during the intervention		647		192	
	Correct recollection		94.0		43.8
	Incorrect recollection		4.0		3.1
	Does not know/remember		2.0		53.1

*Note*: LNS = lipid‐based nutrient supplements.

1 Values are percentage of respondents who mentioned the specific response. Specific responses reported when mentioned by ≥5% of either mothers or fathers.

When asked why a woman might not use supplements during pregnancy, the reasons mothers mentioned included nausea and vomiting (mentioned by 24.2% of mothers), laziness (17.0%), no need (16.7%), the supplements are too costly (6.3%), the supplements cause dizziness (5.6%), and a dislike for medicine and/or supplements (5.3%). Some mothers said they did not know (16.7%), and others said there was no special or particular reason why pregnant women might not use supplements (11.0%). Mothers most commonly cited “no need” as a reason a woman might not use supplements during lactation (30.3%), while 25.7% said they did not know, 20.3% said there was no particular reason, and 9.0% mentioned cost. Again, fathers' perceptions were very similar to maternal perceptions.

When asked why parents might give supplements to their young children, 56.4% of mothers said “to increase the child's appetite,” and 47.3% said “to promote good health.” Other reasons mentioned included to gain weight (13.7%), to help the child become more active (11%), for growth (10.6%), to “get more blood” (9.3%), and to prevent undernutrition (7.6%). Fathers also perceived that parents might give their young children supplements to promote good health (54.5% of fathers) and to increase the child's appetite (44.8%). Mothers said that parents might not give their young children supplements because there was “no need” (37.4%), for no special or particular reason (26.4%), or because they were too costly (15.2%). Fathers also most frequently mentioned that parents might not give their young children supplements because there was “no need” (35.1%).

Finally, mothers and fathers were asked to recall which supplement they received during the intervention. Among mothers in the LNS group, almost all (98.5%) correctly recalled that they had received SQ‐LNS, while 1.2% thought they had received capsules and one respondent could not remember. Among mothers in the IFA or MMN capsules groups, 94.0% correctly remembered that they had received capsules, 4.0% thought they had received SQ‐LNS, and 2.0% could not remember. Fathers, particularly those whose wives were in the capsules group, were less likely to remember which supplement the mother had received. If the mother was in the LNS group, 76.0% of fathers remembered that she had received SQ‐LNS, 2.9% thought she had received capsules, and 21.2% could not remember. If the mother had received capsules, 43.8% of fathers correctly recalled that she had received capsules, 3.1% thought she had received SQ‐LNS, and over half (53.1%) could not remember.

### 3.3 Perceptions of effects of the intervention

Table 3 shows the predicted percentages of mothers who agreed with the various perceptions of the effects of the intervention. Overall, most mothers perceived that the assigned supplements (SQ‐LNS, IFA, or MMN) positively impacted them during pregnancy (89% of mothers) and during lactation (84%), and 90% perceived a positive impact on the index child. Mothers' perceptions of negative impacts were rarer; 17% of mothers perceived that the assigned supplement negatively impacted the mother during pregnancy, 7% perceived negative impacts during lactation, and 7% perceived negative impacts on the index child. With the exception of a marginally significantly higher percentage of mothers in the capsules group reporting that the assigned supplement had a negative impact on the index child (7.9% vs. 4.9%, *p*= .09), there were no significant differences between groups in any of the perceptions. When estimated using the fully adjusted models, these results did not change.

**Table 3:** Maternal perceptions by intervention group

Perception	LNS group		Capsules Group^1^		
	*N*	Predicted percentage (95% CI)^2^	*N*	Predicted percentage (95% CI)^2^	*p* value
1) The assigned supplement had a positive impact on the mother during pregnancy	323	87.4 (83.7, 91.0)	619	89.2 (86.7, 91.6)	.41^3^
2) The assigned supplement had a positive impact on the mother during lactation	311	83.5 (79.7, 87.6)	608	85.1 (82.3, 88.0)	.52^3^
3) The assigned supplement had a negative impact on the mother during pregnancy	332	15.7 (11.7, 19.6)	640	17.0 (14.1, 19.9)	.59^3^
4) The assigned supplement had a negative impact on the mother during lactation	332	6.0 (3.4, 8.5)	638	7.8 (5.7, 9.9)	.29^3^
5) It is acceptable for children aged 6 months and 2 years to be given supplements	307	77.0 (72.2, 81.7)	615	81.2 (78.1, 84.3)	.14^3^
6) The assigned supplement had a positive impact on the index child	306	91.2 (88.0, 94.4)	605	89.3 (86.8, 91.7)	.36^3^
7) The assigned supplement had a negative impact on the index child	332	4.9 (2.5, 7.2)	640	7.9 (5.7, 10.0)	.09^3^
8) There are differences in the health of the index child compared with other children	305	93.9 (91.2, 96.6)	586	92.4 (90.2, 94.5)	.39^3^
9) Supplement's role in child's cognitive development in the future	316		612		IV^4^
Negative impact		1.6		1.3	
No impact		1.9		1.5	
Positive impact		96.5		97.2	
10) Supplement's role in child's performance in school in the future	303		590		0.19^5^
Negative impact		3.6		2.0	
No impact		5.0		4.2	
Positive impact		91.4		93.7	
11) Supplement's role in child's ability to do physical work in the future	314		617		IV^4^
Negative impact		2.2		1.9	
No impact		1.6		2.1	
Positive impact		96.2		96.0	
12) Supplement's role in child's ability to earn money in the future	308		595		IV^4^
Negative impact		2.0		1.0	
No impact		1.3		1.2	
Positive impact		96.7		97.8	
13) The intervention had an impact on other household members	335	56.4 (51.0, 61.8)	631	55.9 (52.0, 59.8)	0.87^3^

*Note*: IFA = iron‐folic acid; LNS = lipid‐based nutrient supplements; MMN = multiple micronutrient.

1 The capsules group is the MMN and IFA capsule groups, combined.

2 Values are percentage of respondents, adjusted for index child age, who indicated they held the specific perception (95% confidence interval).

3 *p* values from logistic regression adjusted for index child age.

4 IV, inadequate variation.

5 *p* values from ordered logistic regression adjusted for index child age.

The most commonly cited positive impacts of the assigned supplement on the mother during pregnancy were improved health (66% of mothers), feeling more hungry (31%), and increased strength or stamina (23%). Commonly cited positive impacts during lactation were improved health (63%), feeling more hungry (20%), and producing more breastmilk (9%). The most commonly cited negative effects during pregnancy were nausea and vomiting (mentioned by 10% of mothers), while the most commonly noted negative impact during lactation was an increased appetite (2%).

Approximately 80% of mothers indicated they believed it was acceptable for children between the ages of 6 months and 2 years to be given supplements. The most commonly mentioned positive effects of the assigned supplement on the index child were that the child was in good health (mentioned by 50% of mothers), that the child was very active (49%), that the child was learning fast (41%), and that the child was growing well (18%). The most commonly reported negative impact was that the child was hyperactive (mentioned by 3% of mothers).

When asked how the current health of the index child compared with other children in the household and other children of similar age, over 90% of mothers indicated that there were differences in the index child's health. While not all differences cited were positive (for example, several mothers mentioned that the child was not growing well or not growing tall), 58% mentioned that the index child was more active than other children, 55% felt the index child was in better health, 44% said the index child was more intelligent than other children, and 9% said the child had healthier skin. When mothers were also asked about their expectations for the supplement's potential impact on the index child's future, over 90% of all mothers, in both intervention groups, expected that the assigned supplement would have a positive impact on the index child's ability to do well in school, her or his cognitive development, her or his ability to do physical work, and her or his ability to earn money.

Finally, when asked to recall whether their participation and the participation of the index child in the intervention had an impact on other household members, approximately 56% of mothers in both groups reported that other household members were impacted by the intervention. The most commonly reported impact was that other household members benefited from the incentives provided by the project, which included things like soap, biscuits, and drinks (44%).

With few exceptions, fathers' perceptions were similar to mothers'. There were no significant intervention group differences in paternal perceptions of the effects of the intervention (Table 4). These results did not change in the fully adjusted models. Like mothers, most fathers perceived a positive impact of the assigned supplement on the mother during pregnancy (approximately 89% of fathers) and lactation (86%), and 97% perceived a positive impact on the index child. Approximately 12% of fathers' perceived negative impacts on the mother during pregnancy, 4% perceived negative impacts on the mother during lactation, and 9% perceived negative impacts on the index child. While approximately 80% of mothers said they felt it was acceptable for children between the ages of 6 months and 2 years to be given supplements, 68% of fathers felt that it was acceptable. The most commonly cited positive impacts reported by fathers were that the child was very active (49% of fathers), was in good health (45%), was learning fast (42%), and was growing well (15%).

**Table 4:** Paternal perceptions by intervention group

Perception	LNS group		Capsules Group^1^		
	*N*	Predicted percentage (95% CI)^2^	*N*	Predicted percentage (95% CI)^2^	*p* value^3^
1) The assigned supplement had a positive impact on the mother during pregnancy	90	89.4 (83.1, 95.8)	166	89.4 (84.6, 94.1)	.99
2) The assigned supplement had a positive impact on the mother during lactation	83	88.1 (81.1, 95.0)	160	84.4 (78.8, 90.0)	.44
3) The assigned supplement had a negative impact on the mother during pregnancy	97	12.3 (5.8, 18.9)	182	12.1 (7.4, 16.8)	.99
4) The assigned supplement had a negative impact on the mother during lactation	98	5.1	184	3.2	IV^4^
5) It is acceptable for children aged 6 months and 2 years to be given supplements	92	69.7 (60.3, 79.1)	174	67.2 (60.3, 74.2)	.68
6) The assigned supplement had a positive impact on the index child	86	100	167	95.8	IV^4^
7) The assigned supplement had a negative impact on the index child	91	6.5 (1.4, 11.6)	175	10.3 (5.8, 14.8)	.31
8) There are differences in the health of the index child compared with other children	93	96.0	177	95.1	IV^4^
9) Supplement's role in child's cognitive development in the future	93		612		IV^4^
Negative impact		2.2		1.7	
No impact		1.0		1.1	
Positive impact		96.8		97.2	
10) Supplement's role in child's performance in school in the future	87		590		IV^4^
Negative impact		1.2		0.6	
No impact		4.6		1.2	
Positive impact		94.3		98.3	
11) Supplement's role in child's ability to do physical work in the future	91		617		IV^4^
Negative impact		0		0	
No impact		0		1.2	
Positive impact		100		98.8	
12) Supplement's role in child's ability to earn money in the future	89		595		IV^4^
Negative impact		0		0	
No impact		0		1.2	
Positive impact		100		98.8	
13) The intervention had an impact on other household members	100	63.0 (53.5, 72.4)	188	61.7 (54.8, 68.7)	0.84

*Note*: IFA = iron‐folic acid; LNS = lipid‐based nutrient supplements; MMN = multiple micronutrient.

1 The capsules group is the MMN and IFA capsule groups combined.

2 Values are percentage of respondents, adjusted for index child age, who indicated they held the specific perception (95% confidence interval).

3 *p* values from logistic regression adjusted for index child age.

4 IV, inadequate variation.

Table S3 shows crosstabulations of the perceived effects of the intervention among households in which both the mother and father were successfully surveyed (*N* = 281). The crosstabulations show that, in general, the perceptions of mothers and fathers in the same household were mostly in agreement. The two perceptions with the highest percentage of disagreement were whether or not the parent felt it was acceptable for children aged 6 months and 2 years to be given supplements (approximately 60% of mother and father pairs disagreed, with more mothers indicating it was acceptable while their husbands did not) and whether the parent perceived that the intervention had an impact on other household members (almost half of mother and father pairs disagreed, with fathers more commonly perceiving impacts on other household members while the mothers did not).

### 3.4 Maternal perceptions related to child consumption of SQ‐LNS

Among mothers in the LNS group, when shown a sachet of SQ‐LNS and asked whether or not the index child received any of the product around the time they turned 6 months of age, 8.7% incorrectly recalled that the index child did not receive SQ‐LNS, and 7.5% could not remember. The descriptive statistics in Table 5 present maternal perceptions related to the index child's consumption of SQ‐LNS among the 83.4% of mothers who recalled that their child received SQ‐LNS.

**Table 5:** Maternal perceptions of the index child's experience with SQ‐LNS (LNS group only)^1^

		Mothers	
		n/N^2^	Percent
(1) Maternal recollection of whether the index child received SQ‐LNS when she/he turned 6 months			
	No	30/346	8.7
	Does not know/remember	26/346	7.5
	Yes	290/346	83.8
(2) There were times when the mother did not give SQ‐LNS to the index child			
	No	179/290	61.7
	Does not know/remember	9/290	3.1
	Yes	102/290	35.2
(2.a) If yes, main reasons for not giving SQ‐LNS to the index child			
	Mother forgot	41/102	40.2
	Child refused food so could not give SQ‐LNS	24/102	23.5
	Child refused food mixed with SQ‐LNS	23/102	22.6
	Travelled	13/102	12.8
(3) Index child's reaction at the end of the intervention			
	Child was indifferent	218/290	75.2
	Does not know/remember	17/290	5.9
	Other reaction	5/290	1.7
	Child asked for SQ‐LNS	50/290	17.2
(3.a) If child asked for SQ‐LNS, frequency that index child asked for SQ‐LNS in the first few weeks after the end of the intervention			
	Less than once a week	13/50	26.0
	About once a week	16/50	32.0
	A few times a week	10/50	20.0
	Every day	9/50	18.0
(4) After the end of the intervention, the mother gave the index child something as a replacement for SQ‐LNS		290	
	No	240/290	82.8
	Does not know/remember	4/290	1.4
	Yes	46/290	15.9
(4.a) If yes, replacements for SQ‐LNS given to index child after the end of the intervention			
	Other supplements	21/46	45.7
	Groundnuts/groundnut paste	7/46	15.2
	Milk	6/46	13.0
	Does not know/remember	7/46	15.2
(5) There was a difference in the foods the index child liked to eat during the intervention compared with other children			
	No	190/290	65.5
	Does not know/remember	11/290	3.8
	Yes	89/290	30.7
(5.a) Observed difference in index child's food preferences during the intervention			
	Preferred food containing groundnuts	61/89	68.5
	Preferred sweet foods/drinks	21/89	23.6
	Refused to eat plain porridge	8/89	9.0
	Preferred rice or rice and stew	6/89	6.7
(6) There was a difference in the foods the index child liked to eat after the end of the intervention compared with other children			
	No	223/290	76.9
	Does not know/remember	8/290	2.8
	Yes	59/290	20.3
(6.a) Observed difference in index child's food preferences after the end of the intervention			
	Preferred food containing groundnuts	30/59	50.9
	Preferred sweet foods/drinks	11/59	18.6
	Preferred rice or rice and stew	8/59	13.6
	Refused to eat plain porridge	7/59	11.9
	Preferred fufu^3^	6/59	10.2

*Note*: LNS = lipid‐based nutrient supplements; SQ‐LNS = Small‐quantity lipid‐based nutrient supplements.

1 Question 1 was asked to all mothers with an index child in the SQ‐LNS group. Questions 2–6 were asked only to mothers who recalled that the index child received SQ‐LNS.

2 For main questions (1–6), values are number of mothers reporting each response or the total number of mothers. For follow‐up questions (2.a–6.a), values are the number of mothers who mentioned the specific response or the total number of mothers in the follow‐up subgroup. Specific subresponses are reported when mentioned by ≥5% of mothers in that subgroup.

3 Fufu is boiled and pounded unripe plantain, cassava, and/or yam, often eaten with soup.

A majority of these mothers (61.7%) said that there was never a time during the intervention period when they did not give the index child SQ‐LNS. Of the 35.2% of mothers who said there were times they did not give the index child SQ‐LNS, the most common reasons were because the mother forgot (40.2%), the child refused food (23.5%), the child refused food specifically mixed with SQ‐LNS (22.6%), and travel (12.8%).

When asked about the index child's reaction when the child stopped receiving SQ‐LNS at the end of the intervention, most mothers reported that the child was indifferent (75.2%), while 17.2% reported that the child asked for SQ‐LNS, and 5.9% could not recall. Among those whose child asked for SQ‐LNS, 18.0% reported that the index child asked for it every day for the first few weeks after supplementation, 20.0% said that the index child requested it a few times a week, 32.0% reported requests approximately once per week, and 26.0% reported that the child requested SQ‐LNS less than once per week.

Most mothers (82.8%) said they did not give the index child anything to replace SQ‐LNS at the end of the intervention. Of the 15.9% who said they used a replacement for SQ‐LNS, 45.7% used another supplement (multivitamins and multivitamin syrups were commonly mentioned), 15.2% said they replaced SQ‐LNS with groundnuts (peanuts) or groundnut paste, and 13.0% said they gave the index child milk (Nido or Vitamilk) as a replacement.

When asked whether they noticed any differences in the foods the index child preferred during the intervention compared with other children, 65.5% said they did not observe any differences. Of the 30.7% who did note differences, 68.5% reported that the index child preferred food that contained groundnuts, 23.6% indicated a preference for sweet food and/or drinks, 9.0% said the child refused to eat plain porridge, and 6.7% reported the child preferred rice or rice with stew (SQ‐LNS was sometimes served to children mixed with porridge or with stew). After the child stopped receiving SQ‐LNS at the end of the intervention, 20.3% of mothers recalled differences in the index child's food preferences compared with other children. Foods containing groundnuts was again the most commonly cited difference in food preferences (50.9%), while a preference for sweet foods or drinks, a preference for rice or rice and stew, refusal to eat plain porridge, and a preference for fufu (boiled and pounded unripe plantain, cassava, and/or yam, often eaten with soup) were also noted.

## 4 DISCUSSION

In semiurban Ghana, both mothers and fathers generally had a positive impression of supplementation, in general, for pregnant and lactating women and for young children. A majority of mothers and fathers in both the LNS group and the capsules group perceived that the assigned supplements, in particular, positively impacted both the mother and the index child, and they expected that the supplements would continue to positively impact the index child's health and human capital into the future. While much rarer, some mothers and fathers did perceive that the intervention had negative impacts on the mother and/or the index child. For both positive and negative impacts, there were no differences in mothers' or fathers' perceptions between intervention groups. This finding is consistent with results from a hypothetical willingness‐to‐pay (WTP) study that was conducted within the same population during the randomised trial. WTP was elicited several times during the trial, and there were no statistically significant differences in WTP for SQ‐LNS for the mother during pregnancy or postpartum or for SQ‐LNS for her infant between the LNS and capsules groups (Adams et al., 2017). Thus, although SQ‐LNS resulted in increased birth size and height of the child at 18 months of age (Adu‐Afarwuah et al., 2015; Adu‐Afarwuah et al., 2016), the differences in birth and growth outcomes may have been too small to be perceptible to parents or may not have been valued enough by parents to trigger relatively more positive perceptions of the impact of SQ‐LNS than of nutrient capsules given only to the mother.

There are several sources of insight into maternal perceptions during the intervention itself (compared with the retrospective or persistent perceptions studied here). During the main trial in Ghana, in‐depth interviews were conducted with a small subset (*n* = 30) of women in the LNS group to explore adherence to study protocol and acceptability of SQ‐LNS for maternal consumption during pregnancy and the first 6 months postpartum (Klevor et al., 2016). Although sensory attributes (taste, texture, smell, etc.) were important for adherence, it was also common for women to cite expected health benefits for themselves or their infants as a motivation for adherence. Attributing nausea and/or vomiting to SQ‐LNS during pregnancy was a commonly mentioned reason for nonadherence during the pregnancy period. We also conducted surveys with all mothers at several time points during the main trial to assess knowledge, attitudes, and practices. Exploratory analysis of select variables from those surveys showed that when the index children were approximately 6 months of age and then again at 18 months, almost all mothers (>98%) across all intervention groups perceived that the index child was growing well. Mothers in the LNS group only were also asked to describe anything they had observed about their child after she or he had been consuming SQ‐LNS for some time. Consistent with the findings from our follow‐up assessment of perceptions, mothers predominately mentioned positive observations (the most commonly cited observations at the child ages of both 6 and 18 months were that the index child was active, eating well or more, gaining weight, sleeping well, and taking more breastmilk). To our knowledge, the only other study to assess perceptions of SQ‐LNS was done in urban Haiti. In focus group discussions conducted during the intervention, mothers were asked what they liked about SQ‐LNS, and mothers indicated that they felt it had positive impacts on their children's growth, health, and development (Lesorogol, Jean‐Louis, Green, & Iannotti, 2015). These perceived positive impacts were also among the most commonly mentioned in Ghana.

This study had several limitations. First, the supplements were provided as part of a randomised controlled efficacy trial, meaning that households did not face any monetary costs to access the supplements, and any time costs were minimal. If households had to regularly travel to a clinic to pick up supplements, for example, or faced other out‐of‐pocket costs to access them, it is not known whether parents' perceptions would be similar to those observed in the context of an efficacy trial. Second, the low rate of successful follow‐up with fathers may have introduced sample selection bias, and therefore all descriptions and analyses of fathers' perceptions are considered exploratory. Another limitation is that there were several small differences in the characteristics of the follow‐up and lost to follow‐up samples, which could have introduced bias, although the differences were small and our results did not change when we controlled for these characteristics. Also, social desirability may have influenced mothers' and fathers' reported perceptions, though the time gap between the intervention and the follow‐up likely lessened this potential issue. Finally, the retrospective nature of some of the questions, which required mothers and fathers to think back over several years, may have introduced recall bias.

Study strengths include a relatively high rate of successful follow‐up among targeted mothers as well as solicitation of both positive and negative perceptions of the intervention and its effect on mothers and their infants. Respondents were purposefully made to feel comfortable about raising either positive or negative perceptions.

The results presented here suggest that similar populations would likely be receptive to programs to deliver SQ‐LNS or micronutrient capsules. The lack of differences between the LNS and capsules groups in perceived benefits of the supplement received indicates that parents viewed all of the nutritional supplements similarly, even though there were demonstrable effects on child growth of SQ‐LNS. This implies that social marketing or other efforts to explain the differences between SQ‐LNS and other supplements might be necessary for programs that include SQ‐LNS. In addition, although perceptions of negative effects were uncommon, educational messages to address these issues, such as nausea and/or vomiting during pregnancy, would be useful. These messages could be delivered in a similar manner as the information provided by study nurses when women complained of negative effects during the intervention, for example, by ensuring that women are aware of common symptoms of pregnancy and providing them with strategies to help cope with them. Maternal accounts of why doses of SQ‐LNS were sometimes skipped could be useful in developing tools and techniques to improve compliance. We conclude that overall, parents in this population generally reflected positively on the iLiNS intervention in Ghana and felt that it had positive and lasting impacts, particularly on the index child, which bodes well for future programmatic efforts to improve maternal and child nutrition in such populations.

## CONFLICTS OF INTEREST

The authors declare that they have no conflicts of interest.

## CONTRIBUTIONS

KGD, SAA, and SAV designed and supervised the parent trial. KGD and SAA supervised the follow‐up study. HO, KPA, MA, BMO, and KGD designed the survey instruments. HO, MEO, and SK coordinated data collection. KPA conducted data analysis. RRY managed data and advised on data analysis. KPA drafted the manuscript, and all authors critically commented on drafts and approved the final manuscript.

## Supporting information

Supplemental Table 1. Nutrient Composition of Supplements
Supplemental Table 2. Characteristics of Maternal Follow‐up Sample by Intervention Group
Supplemental Table 3. Cross Tabulation of Mothers' and Fathers' Perceptions
Supplemental Methods 1. Information and Nutrition Messages. *Information Provided at Enrollment, with Basic Nutrition Message Repeated at 36 Weeks of Gestation*
Supplemental Methods 2. Maternal Perceptions Survey Question. Notes: Italicized text below was read aloud to respondents. Field worker instructions are bracketed. Survey questions are numbered. Nkatepa was the local name for SQ‐LNS.

## References

[mcn12608-bib-0001] Adams, K. P. , Vosti, S. A. , Ayifah, E. , Phiri, T. E. , Adu‐Afarwuah, S. , Maleta, K. , … Dewey, K. G. (2017). Willingness to pay for small‐quantity lipid‐based nutrient supplements for women and children: Evidence from Ghana and Malawi. Maternal & Child Nutrition, *Epub ahead of print*, e12518 10.1111/mcn.12518 28960913 PMC6088232

[mcn12608-bib-0002] Adhvaryu, A. , & Nyshadham, A. (2016). Endowments at birth and parents' investments in children. The Economic Journal, 126(593), 781–820. 10.1111/ecoj.12186 27601732 PMC5010869

[mcn12608-bib-0003] Adu‐Afarwuah, S. , Lartey, A. , Okronipa, H. , Ashorn, P. , Peerson, J. M. , Arimond, M. , … Dewey, K. G. (2016). Small‐quantity, lipid‐based nutrient supplements provided to women during pregnancy and 6 mo postpartum and to their infants fom 6 mo of age increase the mean attained length of 18‐mo‐old children in semi‐urban Ghana: A randomized controlled trial. The American Journal of Clinical Nutrition, 104(3), 797–808. 10.3945/ajcn.116.134692 27534634 PMC4997301

[mcn12608-bib-0004] Adu‐Afarwuah, S. , Lartey, A. , Okronipa, H. , Ashorn, P. , Zeilani, M. , Peerson, J. M. , … Dewey, K. G. (2015). Lipid‐based nutrient supplement increases the birth size of infants of primiparous women in Ghana. The American Journal of Clinical Nutrition, 101(4), 835–846. 10.3945/ajcn.114.091546 25833980

[mcn12608-bib-0005] Adu‐Afarwuah, S. , Lartey, A. , Zeilani, M. , & Dewey, K. G. (2011). Acceptability of lipid‐based nutrient supplements (LNS) among Ghanaian infants and pregnant or lactating women. Maternal & Child Nutrition, 7(4), 344–356. 10.1111/j.1740-8709.2010.00286.x 21496207 PMC6860738

[mcn12608-bib-0006] Almond, D. , & Mazumder, B. (2013). Fetal origins and parental responses. Annual Review of Economics, 5(1), 37–56. 10.1146/annurev-economics-082912-110145

[mcn12608-bib-0007] Ashorn, P. , Alho, L. , Ashorn, U. , Cheung, Y. B. , Dewey, K. G. , Gondwe, A. , … Maleta, K. (2015a). Supplementation of maternal diets during pregnancy and for 6 months postpartum and infant diets thereafter with small‐quantity lipid‐based nutrient supplements does not promote child growth by 18 months of age in rural Malawi: A randomized controlled trial. The Journal of Nutrition, 145(6), 1345–1353. 10.3945/jn.114.207225 25926413

[mcn12608-bib-0008] Ashorn, P. , Alho, L. , Ashorn, U. , Cheung, Y. B. , Dewey, K. G. , Harjunmaa, U. , … Maleta, K. (2015b). The impact of lipid‐based nutrient supplement provision to pregnant women on newborn size in rural Malawi: A randomized controlled trial. The American Journal of Clinical Nutrition, 101(2), 387–397. 10.3945/ajcn.114.088617 25646337

[mcn12608-bib-0009] Ashorn, U. , Alho, L. , Arimond, M. , Dewey, K. G. , Maleta, K. , Phiri, N. , … Ashorn, P. (2015c). Malawian mothers consider lipid‐based nutrient supplements acceptable for children throughout a 1‐year intervention, but deviation from user recommendations is common. The Journal of Nutrition, 145(7), 1588–1595. 10.3945/jn.114.209593 25995276

[mcn12608-bib-0010] Dewey, K. G. , Mridha, M. K. , Matias, S. L. , Arnold, C. D. , Cummins, J. , Khan, M. S. A. , … Vosti, S. A. (2017). Lipid‐based nutrient supplementation in the first 1000 d improves child growth in Bangladesh: A cluster‐randomized effectiveness trial. American Journal of Clinical Nutrition, 105(4), 944–957. 10.3945/ajcn.116.147942 28275125

[mcn12608-bib-0011] Ghana Statistical Service, Ghana Health Service, & ICF International (2015). Ghana demographic and health survey 2014. Retrieved from Rockville, Maryland, USA:

[mcn12608-bib-0012] Hess, S. Y. , Abbeddou, S. , Jimenez, E. Y. , Somé, J. W. , Vosti, S. A. , Ouédraogo, Z. P. , … Brown, K. H. (2015). Small‐quantity lipid‐based nutrient supplements, regardless of their zinc content, increase growth and reduce the prevalence of stunting and wasting in young burkinabe children: A cluster‐randomized trial. PLoS One, 10(3), e0122242 10.1371/journal.pone.0122242 25816354 PMC4376671

[mcn12608-bib-0013] Iannotti, L. L. , Dulience, S. J. L. , Green, J. , Joseph, S. , François, J. , Anténor, M.‐L. , … Nickerson, N. M. (2014). Linear growth increased in young children in an urban slum of Haiti: A randomized controlled trial of a lipid‐based nutrient supplement. The American Journal of Clinical Nutrition, 99(1), 198–208. 10.3945/ajcn.113.063883 24225356 PMC3862455

[mcn12608-bib-0014] Klevor, M. K. , Adu‐Afarwuah, S. , Ashorn, P. , Arimond, M. , Dewey, K. G. , Lartey, A. , … Ashorn, U. (2016). A mixed method study exploring adherence to and acceptability of small quantity lipid‐based nutrient supplements (sq‐LNS) among pregnant and lactating women in Ghana and Malawi. BMC Pregnancy and Childbirth, 16(1), 253 10.1186/s12884-016-1039-0 27577112 PMC5004276

[mcn12608-bib-0015] Lesorogol, C. , Jean‐Louis, S. , Green, J. , & Iannotti, L. (2015). Preventative lipid‐based nutrient supplements (LNS) and young child feeding practices: Findings from qualitative research in Haiti. Maternal & Child Nutrition, 11, 62–76. 10.1111/mcn.12122 24784976 PMC6860279

[mcn12608-bib-0016] Maleta, K. M. , Phuka, J. , Alho, L. , Cheung, Y. B. , Dewey, K. G. , Ashorn, U. , … Ashorn, P. (2015). Provision of 10–40 g/d lipid‐based nutrient supplements from 6 to 18 months of age does not prevent linear growth faltering in Malawi. The Journal of Nutrition, 145(8), 1909–1915. 10.3945/jn.114.208181 26063066

[mcn12608-bib-0017] Mridha, M. K. , Matias, S. L. , Chaparro, C. M. , Paul, R. R. , Hussain, S. , Vosti, S. A. , … Dewey, K. G. (2016). Lipid‐based nutrient supplements for pregnant women reduce newborn stunting in a cluster‐randomized controlled effectiveness trial in Bangladesh. The American Journal of Clinical Nutrition, 103(1), 236–249. 10.3945/ajcn.115.111336 26607935 PMC6443293

[mcn12608-bib-0018] Phuka, J. C. , Maleta, K. , Thakwalakwa, C. , Chenug, Y. B. , Briend, A. , Manary, M. J. , & Ashorn, P. (2008). Complementary feeding with fortified spread and incidence of severe stunting in 6‐ to 18‐month‐old rural Malawians. Archives of Pediatrics & Adolescent Medicine, 162(7), 619–626. 10.1001/archpedi.162.7.619 18606932 PMC3721756

[mcn12608-bib-0019] Prado, E. L. , Adu‐Afarwuah, S. , Lartey, A. , Ocansey, M. , Ashorn, P. , Vosti, S. A. , & Dewey, K. G. (2016). Effects of pre‐ and post‐natal lipid‐based nutrient supplements on infant development in a randomized trial in Ghana. Early Human Development, 99, 43–51. 10.1016/j.earlhumdev.2016.05.011 27391572

