# Supplementary material for: Ghanaian parents' perceptions of pre and postnatal nutrient supplements and their effects
Source: Matern Child Nutr. 2018 Apr 15;14(4):e12608. doi: 10.1111/mcn.12608 (PMC6866179; doi:10.1111/mcn.12608)
Supplement: Supplementary file 1 — Supplemental Table 1. Nutrient Composition of Supplements Supplemental Table 2. Characteristics of Maternal Follow‐up Sample by Intervention Group Supplemental Table 3. Cross Tabulation of Mothers' and Fathers' Perceptions Supplemental Methods 1. Information and Nutrition Messages. Information Provided at Enrollment, with Basic Nutrition Message Repeated at 36 Weeks of Gestation Supplemental Methods 2. Maternal Perceptions Survey Question. Notes: Italicized text below was read aloud to respondents. Field worker instructions are bracketed. Survey questions are numbered. Nkatepa was the local name for SQ‐LNS. [file MCN-14-e12608-s001.pdf]

Online Supporting Material: Ghanaian Parents' Perceptions of Pre- and Post-Natal Nutrient Supplements and their Effects

Supplemental Methods

Supplemental Methods 1. Information and Nutrition Messages

Information Provided at Enrollment, with Basic Nutrition Message Repeated at 36 Weeks of Gestation

To women who received capsules:

1. The capsules are all for you because women need more vitamins and minerals when they are pregnant.
2. You will have to take one capsule per day, every day in the week.
3. Do not share the capsules with others.
4. You will take the capsule with water in the morning after you have eaten. If you forget to take it in the morning, then take it in the afternoon after you have eaten. If you forget it in the morning and also in the afternoon, then take it in the evening after you have eaten. If you forget to take the capsule during the whole day, do not take two capsules the next day; it is always one capsule per day.
5. If you need to travel for a number of days, take the capsules with you so you can take them every day, whilst you are away.
6. Do not forget to eat meat, fish, eggs, fruits and vegetables whenever you can. You still need these foods even if you take the capsules we have given you.

To women who received Nkatepa (local name for SQ-LNS):

1. This Nkatepa supplement is all for you because women need more vitamins and minerals when they are pregnant.
2. You will have to eat one sachet per day, every day in the week.
3. Do not share the Nkatepa with others.
4. Mix the entire content of one sachet of Nkatepa with one ladle of food (any food you want) in the morning and eat. The one ladle of food is to make sure that you eat the amount of supplement you need for the day and not leave some of it mixed with food behind and let it go to waste. When that happens, it means you did not eat the amount of supplement your body needs for the day.
5. After you have eaten the one ladle of food mixed with the supplement, you can then go ahead and eat more of your food.
6. If you forget to eat the supplement in the morning, then eat it in the afternoon. If you forget it in the morning and also in the afternoon, then eat it in the evening.
7. If you forget the supplement the whole day, do not eat two sachets the next day; it is always one sachet per day.
8. If you need to travel for a number of days, take the Nkatepa with you so you can take them every day, whilst you are away.
9. Do not forget to eat meat, fish, eggs, fruits and vegetables whenever you can. You still need these foods even if you eat the Nkatepa we have given you.

Information Provided at 6 Months Postpartum

To women whose infants received no supplementation:

1. Breastfeed your baby as you did before.
2. Please do not forget to give your baby other things such as eggs, fruits and vegetables whenever you can. Your baby still needs these foods.

To women whose infants received Nkatepa (local name for SQ-LNS):

1. Breastfeed your baby as you did before.
2. This Nkpatepa supplement is for your baby because babies need special foods from 6 months of age; do not share it with others.
3. The baby will need to eat two (2) sachets per day, every day in the week. That is, you will give one sachet in the morning and another sachet in the afternoon or evening.
4. Each time you are giving the supplement to your baby, here is what you will do:
    - You will mix the entire content of the sachet with 2-3 tablespoons of already prepared food and feed it to the baby.
    - After the baby has eaten the 2-3 tablespoons mixed with the supplement, you can go ahead and give him/her more of the food. The 2-3 tablespoons is to make sure that the baby eats all of the supplement in the sachet and not leave some of it mixed with food behind and let it go to waste.
    - You can mix the Nkatepa with any food you are giving to the baby.
5. Do not cook food with the supplement; store the supplement at room temperature; you do not need to keep the supplement in the refrigerator.
6. If one day you did not give the supplement to your baby at all, or gave only one sachet instead of two sachets, do not give more than two sachets the next day; it is always two sachets per day.
7. In case you need to travel with the baby for a number of days, take the Nkatepa with you so you can give it to the baby every day, whilst you are away.
8. Do not forget to give your baby other things such as eggs, fruits and vegetables whenever you can. You baby still needs these foods even if you give him/her Nkatepa

Supplemental Methods 2. Maternal Perceptions Survey Questions

Notes: Italicized text below was read aloud to respondents. Field worker instructions are bracketed. Survey questions are numbered. Nkatepa was the local name for SQ-LNS.

Some women take in supplements when they are pregnant. Others do not take in any supplements. We would like to ask you some questions about women's supplement use during pregnancy and lactation. When I say "supplement" I mean products that you might get from a hospital, clinic or pharmacy, like vitamin and mineral supplements. Here are some examples (show examples).

[FW: Do not read out responses to questions. Allow the woman to mention all her responses and tick all that apply]

1. In your opinion, what are all the different reasons a woman might <u>use supplements</u> during pregnancy?
2. How about during lactation? Please share with me all the different reasons a mother might use supplements during lactation?
3. Thinking back to pregnancy again, in your opinion, what are the reasons a woman might <u>not use supplements</u> during pregnancy?
4. And during lactation? What are some reasons a mother might not use supplements during lactation?

I would now like to ask you some questions about giving supplements to children between 6 mo and 2 years. When I say "supplement" I mean products that you might get from a hospital, clinic or pharmacy, like vitamin and mineral supplements. Here are some examples (show examples of some supplements given to children).

5. Do you think it is ok for children who are between 6 mo and 2 years to be given supplements?

[FW: Do not read out responses to questions. Allow the woman to mention all her responses and tick all that apply]

6. In your opinion, what are the reasons mothers may give supplements to their children aged between 6 mo and 2 years?
7. In your opinion, what are the reasons mothers may not give supplements to their children aged between 6 mo and 2 years?

Now we would like to ask you some questions about your experiences regarding the iLiNS project. We are interested in both positive and negative experiences. Please think back to when you were a participant in this project and feel free to share with us whatever your experiences were, whether good or bad. Remember, it's ok to share both.

[FW: Show pictures of Nkatepa and Capsule]

8. Which supplements did you receive from the iLiNS project when you were pregnant with and/or breastfeeding [INDEX CHILD]

I would like to ask you about any impact on you yourself of the [Nkatepa/ capsule] that you took during pregnancy and/ or lactation. I would like to ask about both good and bad impacts. When I say "impact" I mean any changes that you experienced that you think were because of the [Nkatepa/capsule].

[FW: Do not read out responses to questions. Allow the woman to mention all her responses and tick all that apply]

9. Can you share with me any negative impact on you yourself that was due to the [Nkatepa/ Capsule] that you took during pregnancy?
10. How about during lactation? Can you share with me any negative impact on you yourself that was due to the [Nkatepa/ Capsule] that you took during lactation?
11. Can you share with me any positive impact on you yourself that was due to the [Nkatepa/ Capsule] that you took during pregnancy?
12. Can you share with me any positive impact on you yourself that was due to the [Nkatepa/ Capsule] that you took during lactation?

I would like to ask you about any impact on your child of the [Nkatepa/ capsule] that you and/ or your child took during the iLiNS project. I would like to ask about both good and bad impacts. When I say "impact" I mean any changes that you observed in your child that you think was because of the [Nkatepa/capsule].

13. Did you observe any negative impact on your child that you think was due to the [Nkatepa/ Capsule] that you/ your child took?

[FW: Do not read out responses to questions. Allow the woman to mention all her responses and tick all that apply]

14. What type of negative impacts do you think were due to the [Nkatepa/ Capsule] that you/ your child took?

[FW: Q15 applies to LNS group only (if woman answered 'Nkatepa" to Q8)]

15. Do you think the negative impacts were due to the Nkatepa you took or due to the Nkatepa your child took or both?
16. Did you observe any positive impact on your child that you think was due to the [Nkatepa/ Capsule] that you/ your child took?
17. What type of positive impacts do you think were due to the [Nkatepa/ Capsule] that you/ your child took?

[FW: Q18 applies to LNS group only (if woman answered 'Nkatepa" to Q8)**]**

18. Do you think the positive impacts were due to the Nkatepa you took or due to the Nkatepa your child took or both?

I would now like to ask you some questions about how your participation in the iLiNS project impacted other members of your household. When I say "impact" I mean any changes that you think other

members of your household experienced because of your participation in the project. Please remember that the changes can be positive or negative.

[FW: Do not read out responses to questions. Allow the woman to mention all her responses and tick all that apply]

19. In what ways did your and [index child's] participation in the iLiNS study impact other member (s) of your household?

Now, we know that all children have the potential to grow, learn, develop, work and earn money in the future. But the ability to achieve these differs from child to child. Now I want you to think about other children in your household or other children you know. I would like you to compare [INDEX CHILD] with these other children in your response to my questions.

20. When you compare [index child] to other children in your household or other children around [index child's] age, do you observe any differences in health?

21. Can you please tell me some of the differences that you observe in the health of [index child] compared to other children?

*Children's health and future may be influenced by different things. Supplements women and children take in may or may not play a role in children's futures. Now I am going to mention different aspects of children's futures and I will like to know if in your opinion, the supplement <u>you and/or your child took as part of the iLiNS Project,</u> impacts those areas of the [INDEX CHILD'S] future. If you do not think the supplement you and/or your child took as part of the iLiNS Project plays a role, please feel free to tell me that in your response. Please remember that w*hen I say "impact" I mean any changes that you think [INDEX CHILD] experienced because of his/her participation in the project.

[FW: For each of the questions below, please **<u>read out the first three responses (no impact, positive impact, negative impact)</u>** to mother and choose only one response. Remember to repeat the first 3 responses for each question]

22. *What is your opinion regarding the supplement's role in:*
    a. [INDEX CHILD'S] performance in school
    b. [INDEX CHILD'S] cognitive development *(When I say cognitive development, I mean getting better at remembering things and figuring things out, reasoning, and problem solving.)*
    c. [INDEX CHILD'S] ability to do physical work
    d. [INDEX CHILD'S] ability to earn money as an adult

<u>For LNS group only</u>**:** *You and your child received Nkatepa for a period while you were in the iLiNS project. Now I am going to ask you some questions regarding the Nkatepa you and your child took. I will also ask about the time right after your child stopped receiving Nkatepa.*

23. Did [INDEX CHILD] receive any Nkatepa from the iLiNS project when they turned 6 mo? Here is an example [show a sample of Nkatepa].

[FW: Do not read out responses to questions. Allow the woman to mention all her responses and tick all that apply]

24. Were there times when you did not give Nkatepa to your child?
25. At times when you did not give Nkatepa to your child, what were the main reasons?
26. When your child's participation in the iLiNS study ended and you no longer received Nkatepa to give to your child, how did your child react?

[FW: For Q27 only, please **read out the first four responses (less than once a week, about once a week, a few times a week, every day)** to mother and choose only one response.

27. Thinking back to the first few weeks after you stopped receiving Nkatepa for your child, how often would you say your child asked for it?
28. After you stopped receiving Nkatepa for your child, did you give anything to your child as a replacement?
29. Can you please tell me what you gave to your child as a replacement after you stopped receiving Nkatepa for your child?
30. In comparison to other children, did you see any differences in the foods the child liked to eat during the time s/he received Nkatepa?
31. Compared to other children, what were some of the differences you observed in the foods your child liked to eat during the time s/he received Nkatepa?
32. How about after s/he stopped receiving Nkatepa? Did you see any differences in the foods your child liked to eat?
33. What were some of the differences you observed in the foods your child liked to eat after s/he stopped receiving Nkatepa, compared to other children?

Supplemental Tables

Supplemental Table 1. Nutrient Composition of Supplements

	Nutrient Content per Daily Ration			
	Infant LNS[1]	Maternal LNS[1]	Multiple Micronutrient Capsule	Iron-Folic Acid Capsule
Daily Ration (g/day)	20	20		
Total energy (kcal)	118	118		
Protein (g)	2.6	2.6		
Fat (g)	9.6	10		
Linoleic acid (g)	4.46	4.59		
α-Linoleic acid (g)	0.58	0.59		
Vitamin A (µg RE)	400	800	800	
Vitamin C (mg)	30	100	100	
Vitamin B_1 (mg)	0.3	2.8	2.8	
Vitamin B_2 (mg)	0.4	2.8	2.8	
Niacin (mg)	4	36	36	
Folic acid (mg)	80	400	400	400
Pantothenic acid (mg)	1.8	7	7	
Vitamin B_6 (mg)	0.3	3.8	3.8	
Vitamin B_{12} (µg)	0.5	5.2	5.2	
Vitamin D (IU)	200	400	400	
Vitamin E (mg)	6	20	20	
Vitamin K (µg)	30	45	45	
Iron (mg)	6	20	20	60
Zinc (mg)	8	30	30	
Cu (mg)	0.34	4	4	
Calcium (mg)	280	280		
Phosphorus (mg)	190	190		
Potassium (mg)	200	200		
Magnesium (mg)	40	65		
Selenium (µg)	20	130	130	
Iodine (µg)	90	250	250	
Manganese (mg)	1.2	2.6	2.6	

[1]Nutrient contents for infant and maternal LNS include contributions from the food ingredients (vegetable oil, groundnut paste, and powdered milk) as well as from the multiple micronutrient premix.
Sources: (Adu-Afarwuah et al., 2016; Adu-Afarwuah et al., 2015)

Supplemental Table 2. Characteristics of Maternal Follow-up Sample by Intervention Group

Variable	LNS Group		Capsules Group		P-value
	N	Mean ± SD	N	Mean ± SD	
Index child age at maternal perceptions data collection (y)	335	5.0 ± 0.6	645	4.9 ± 0.6	0.13
Index child male (%)	335	49	646	48	0.81
Maternal parity at pregnancy with index child (n)	335	2.3 ± 1.3	646	2.3 ± 1.3	0.41
Maternal age (y)	334	27.1 ± 5.5	646	26.9 ± 5.4	0.50
Maternal education (y)	334	7. 4 ± 3.8	644	7.4 ± 3.6	0.91
Maternal height (m)	335	1.6 ± 0.05	645	1.6 ± 0.06	0.56
Head of household female (%)	334	23	642	27	0.17
Household has electricity (%)	334	84	644	87	0.29

Notes: Values are mean ± standard deviation for continuous variables and percentage for dichotomous variables. P-values for tests of difference in mean/percentage between intervention groups from logistic (for dichotomous variables) and OLS (for continuous variables) regressions.

Supplemental Table 3. Cross Tabulation of Mothers' and Fathers' Perceptions

			Fathers		
1) The assigned supplement had a positive impact on the mother during pregnancy			No	Yes	
	**Mothers**	No	1	12	
		Yes	25	195	
			**Fathers**		
2) The assigned supplement had a positive impact on the mother during lactation			No	Yes	
	**Mothers**	No	5	13	
		Yes	28	172	
			**Fathers**		
3) The assigned supplement had a negative impact on the mother during pregnancy			No	Yes	
	**Mothers**	No	198	20	
		Yes	36	9	
			**Fathers**		
4) The assigned supplement had a negative impact on the mother during lactation			No	Yes	
	**Mothers**	No	244	5	
		Yes	11	5	
			**Fathers**		
5) It is acceptable for children aged 6 months and 2 years to be given supplements			No	Yes	
	**Mothers**	No	16	23	
		Yes	60	145	
			**Fathers**		
6) The assigned supplement had a positive impact on the index child			No	Yes	
	**Mothers**	No	1	12	
		Yes	6	208	
			**Fathers**		
7) The assigned supplement had a negative impact on the index child			No	Yes	
	**Mothers**	No	211	19	
		Yes	11	3	
			**Fathers**		
8) There are differences in the health of the index child compared to other children			No	Yes	
	**Mothers**	No	2	10	
		Yes	10	213	
				**Fathers**	
9) Supplement's role in child's cognitive development in the future			Negative	No Impact	Positive
	**Mothers**	Negative	0	0	0
		No impact	0	1	3
		Positive	4	2	246
				**Fathers**	
10) Supplement's role in child's performance in school in the future			Negative	No Impact	Positive
	**Mothers**	Negative	0	0	2
		No impact	0	0	8
		Positive	1	5	224

			Fathers		
			Negative	No Impact	Positive
11) Supplement's role in child's ability to do physical work in the future	**Mothers**	Negative	0	0	3
		No impact	0	0	5
		Positive	3	3	240
			**Fathers**		
			Negative	No Impact	Positive
12) Supplement's role in child's ability to earn money in the future	**Mothers**	Negative	0	0	1
		No impact	0	0	3
		Positive	0	1	233
			**Fathers**		
			No	Yes	
13) The intervention had an impact on other household members	**Mothers**	No	52	97	
		Yes	48	126	
